# Patient Safety Implications of Opportunistic Pathogens and Healthcare-Associated Infections in COVID-19 Patients: A Narrative Review

**DOI:** 10.3390/healthcare14121614

**Published:** 2026-06-08

**Authors:** Francesco De Micco, Gianmarco Di Palma, Davide Ferorelli, Flavia Giacomobono, Isabella Lima Arrais Ribeiro, Johnys Berton Medeiros da Nóbrega, Roberto Scendoni

**Affiliations:** 1 Research Unit of Bioethics and Humanities, Department of Medicine and Surgery, Università Campus Bio-Medico di Roma, Via Alvaro del Portillo, 00128 Roma, Italy; f.demicco@policlinicocampus.it (F.D.M.); g.dipalma@policlinicocampus.it (G.D.P.); 2 Operative Research Unit of Department of Clinical Affairs, Fondazione Policlinico Universitario, 00128 Roma, Italy; 3 Interdisciplinary Department of Medicine (DIM), Section of Legal Medicine, University of Bari “Aldo Moro”, 70121 Bari, Italy; davide.ferorelli@uniba.it; 4 Postgraduate Program in Dentistry, Federal University of Paraíba, Campus I, João Pessoa 58051-900, Brazil; isabella_arrais@yahoo.com (I.L.A.R.); johnysberton@gmail.com (J.B.M.d.N.); 5 Department of Law, Institute of Legal Medicine, University of Macerata, 62100 Macerata, Italy; r.scendoni@unimc.it

**Keywords:** healthcare-associated infections, multidrug-resistant pathogens, antimicrobial stewardship, patient safety

## Abstract

The COVID-19 pandemic has highlighted the increased vulnerability of hospitalized patients to healthcare-associated infections (HAIs), which significantly impact patient safety and clinical outcomes. This narrative review summarizes the main opportunistic pathogens associated with HAIs in COVID-19 patients, with particular focus on multidrug-resistant organisms such as *Klebsiella pneumoniae*, *Pseudomonas aeruginosa*, *Acinetobacter baumannii*, *Staphylococcus aureus*, and *Candida* spp. The review also examines key aspects of antimicrobial resistance, prevention and control strategies, and medico-legal implications. The evidence supports the need for a multifaceted approach based on antibiotic stewardship, infection prevention guidelines, and multidisciplinary clinical risk management.

## 1. Introduction

Healthcare-associated infections (HAIs) represent a global issue, with a significant impact on mortality, length of hospital stay, and healthcare costs. According to data from the European Centre for Disease Prevention and Control (ECDC), it is estimated that millions of patients in Europe contract an infection each year during hospitalization. In Italy, HAIs affect approximately 5–8% of hospitalized patients, with an even higher rate in intensive care units. Healthcare-associated infections (HAIs) are 2–4 times more prevalent in low-income countries (LICs) and low- and middle-income countries (LMICs) compared to high-income countries, with overall prevalence rates of 12.9–15.5% in resource-limited settings versus 3.2–7.1% in the United States and Europe [1,2].

The disparity is even more pronounced in intensive care units and for device-associated infections.

The COVID-19 pandemic has exacerbated the risk of healthcare-associated infections due to increased hospitalizations, intensive care stays, and the widespread use of invasive procedures and immunosuppressive therapies. In severe cases, the combination of SARS-CoV-2-induced lung damage and secondary bacterial or fungal colonization further worsens clinical outcomes. This article presents a narrative review of opportunistic pathogens in COVID-19 patients, focusing on risk factors, resistance mechanisms, prevention strategies, and medico-legal implications. A comprehensive literature search was performed across PubMed, Scopus, and Web of Science. The literature search strategy was based on a combination of keywords referring to COVID-19 (“COVID-19”, “SARS-CoV-2”), healthcare-associated infections (“healthcare-associated infections”, “nosocomial”, “coinfection”, “superinfection”), and antimicrobial resistance (“antimicrobial resistance”, “multidrug-resistant”, “resistance”). The search timeframe was set from December 2019 to January 2026, aligning with the emergence of SARS-CoV-2, to ensure a comprehensive inclusion of studies addressing healthcare-associated infections in the context of COVID-19. The database search identified a total of 973 records. After duplicate detection and resolution, 663 duplicate records were identified, leaving 310 unique records. Articles were considered relevant if they addressed healthcare-associated, nosocomial, secondary, or opportunistic infections in COVID-19 patients, particularly in relation to risk factors, antimicrobial resistance, multidrug-resistant organisms, infection prevention and control strategies, clinical outcomes, or patient-safety implications. Only articles published in peer-reviewed journals, systematic reviews, and high-impact conference proceedings were considered, while conference abstracts and publications in languages other than English were excluded. The included studies were critically appraised based on their methodological robustness, consistency of findings, and relevance to the research question.

In order to ensure the methodological rigor of this narrative review, the SANRA (Scale for the Assessment of Narrative Review Articles) criteria were applied. SANRA is a validated six-item tool designed to assess the quality of narrative reviews, focusing on the following dimensions: justification of the article, clarity of stated objectives, appropriateness of literature sources, quality of data presentation, depth of scientific reasoning, and relevance to clinical practice. Given the narrative nature of this study, SANRA was employed to support transparency and consistency in the synthesis of literature and the discussion of findings.

## 2. Types of Healthcare-Associated Infections

Healthcare-associated infections (HAIs) are frequent and potentially life-threatening hospital complications. Among the most significant are ventilator-associated pneumonia (VAP), central line-associated bloodstream infections (CLABSIs), catheter-associated urinary tract infections (CAUTIs), and surgical site infections (SSIs). Opportunistic pathogens could be defined as microorganisms that primarily cause infections in immunocompromised or critically ill patients, some of them may also be notable for antimicrobial resistance or frequent occurrence in hospital settings [3].

Indeed, reported rates of VAP in COVID-19 patients admitted to ICUs and undergoing mechanical ventilation reach up to 45%, with prevalence ranging from 37.8% to 53.2% according to main clinical and epidemiological studies [4,5]. The percentage is significantly higher compared to non-COVID patients (approximately 23–40%), which is a threefold risk increase in the situation of SARS-CoV-2 infection [5,6]. VAP is also frequently associated with higher hospital mortality, which is compromised in about 43–45% of patients [5]. Recent data also report an incidence of nosocomial pneumonia (NP) of 14–30% in COVID-19 patients, with a VAP rate of up to 14 per 1000 ventilator days and mortality rates as high as 64% compared to 16% in non-COVID patients [5].

CLABSI rates have been reported to increase during the pandemic. Studies in the US and Saudi Arabia reported increases ranging from 16% to 51% in standardized central line-associated bloodstream infection rates in 2020 compared to 2019 [7]. For instance, in a Saudi Arabian network of 78 hospitals, the rate went up from 2.16 to 2.75 CLABSI events per 1000 central-line days, reaching its peak in 2020 [7]. This rise can be attributed to staff shortages, shifts in care practice, and routine administration of immunosuppressive treatments [7].

CAUTI rates have been reported to decrease: catheter-associated urinary tract infections decreased by approximately 36% during the pandemic (from 1.54 to 0.96 events per 1000 catheter days) [7]. This can be attributed to fewer catheter insertions, more stringent protocols, or the overall decrease in non-emergency hospital admissions. The reported reduction in CAUTI is further supported by other literature sources; however, to date, no systematic reviews or meta-analyses are available to strengthen the validity of this hypothesis [7,8].

Regarding surgical site infections (SSIs), while less documented in the COVID-19 context, the available evidence suggests an increase, due in part to the compromised clinical condition of patients and emergency surgery, without, however, quantitative data being abundant [4].

These findings highlight the importance of reinforcing infection prevention and control measures—such as hand hygiene, appropriate catheter and ventilator care, microbiological surveillance, and sanitation procedures—alongside continuous staff training. While these practices are essential in any healthcare setting, the COVID-19 pandemic has particularly highlighted their critical role in mitigating hospital-acquired infections and associated mortality.

## 3. Opportunistic and Multidrug-Resistant Pathogens

During the COVID-19 pandemic, hospitalized patients have shown increased susceptibility to opportunistic pathogens, each associated with specific clinical and resistance-related challenges due to their virulence and resistance profiles. *Klebsiella pneumoniae*, and more broadly carbapenemase-producing strains (CRKPs), including other species rose significantly: a European hospital reported a nearly fivefold increase, from 0.18% before the pandemic to 0.76% during it, with nearly half in SARS-CoV-2-positive patients, predominantly in ICU ventilated patients [9,10]. These CRKP infections tend to evolve into ventilator-associated pneumonia (VAP) and bloodstream infections, driving case-fatality rates as high as 65% [11,12].

*Pseudomonas aeruginosa* represents another relevant pathogen, which thrives in ICU settings by forming biofilms on endotracheal tubes, a feature present in over 90% of intubated patients. The resistance of this bacterial agent in biofilmic contexts is the result of several molecular patterns, including the production of β-lactamases, enzymes capable of blocking antibiotics of the β-lactam category, the availability of efflux pumps such as the MexAB-OprM and MexXY-OprM systems capable of throwing drugs out of the bacterial cell and reducing their internal concentration and therapeutic efficacy and, finally, a key role is played by the second messenger c-di-GMP, which is able to upregulate the genes involved in the transition between the free form, also called planktonic, and the one organized in biofilms, thus modulating antibiotic resistance [13,14,15].

The pathogen has been reported as a common cause of late-onset VAP in COVID-19 patients, with reported mortality rates of approximately 42% in the presence of biofilms [16,17,18,19].

Another key opportunistic bacterial pathogen is *Acinetobacter baumannii*, often resistant to carbapenems and colistin. ICUs worldwide saw infection rates nearly doubling during COVID-19, with odds ratios around 1.9 in one large study [20]. This pathogen’s ability to form resilient biofilms further complicates disinfection efforts and limits treatment options.

Among Gram-positive threats, methicillin-resistant *Staphylococcus aureus* (MRSA) remains a major cause of severe secondary infections—such as pneumonia and sepsis—in COVID-19 patients who are ventilated. Its resistance through biofilm formation allows it to evade both immune system function and traditional antibiotics [17,21,22]. Studies conducted during the COVID-19 pandemic also showed that MRSA colonization among hospitalized patients was high, especially for those admitted to intensive care, suggesting that this stay may have led to an increase in MRSA infections. Patients colonized by this bacterial agent represent real reservoirs for transmission, highlighting the importance of active surveillance and infection control measures even in pandemic contexts [23,24].

Fungal pathogens have also emerged with foreboding prominence. *Candida* spp., in particular the multidrug-resistant *Candida auris*, have been responsible for outbreaks in ICU catheter-associated infections, with candidemia mortality ranging from 30% to as high as 80% (mdpi.com). *Aspergillus* spp., in particular *A. fumigatus*, meanwhile, has been the cause of invasive pulmonary aspergillosis in critically ill COVID-19 patients; an international cohort reported a mortality of >50%, with *A. fumigatus* responsible for >80% of isolates [25,26]. In addition, numerous cases of invasive mucormycosis (also known as zygomycetes infections) have been identified in patients with COVID-19, particularly in subjects also affected by diabetes or undergoing corticosteroid-based therapies; this disease is characterized by high morbidity and mortality [24].

Collectively, these opportunistic pathogens—marked by multidrug resistance, biofilm production, and increased virulence—have introduced complexity to patient management throughout the pandemic. To effectively address these infections, health systems should adopt accelerated microbiological diagnostics, strict antimicrobial stewardship, vigorous infection control practices, and intense surveillance, particularly in ICU environments where the risk of transmission and adverse outcomes remains highest [27,28,29,30,31,32].

## 4. Mechanisms of Antimicrobial Resistance

Among the key challenges to the treatment of healthcare-associated infections (HAIs) during the COVID-19 pandemic is the multifaceted nature of antimicrobial resistance mechanisms exhibited by opportunistic pathogens. Perhaps the first line of defense for most bacteria is the elaboration of inactivating enzymes. For example, beta-lactamases such as extended-spectrum (ESBLs) and carbapenemases are able to hydrolyze penicillins, cephalosporins, and even carbapenems. *Acinetobacter baumannii* and *Klebsiella pneumoniae*, for instance, use class D OXA-type enzymes that significantly reduce the effectiveness of antibiotics by degrading such drug molecules directly prior to their action [33,34].

Cooperating with enzyme defenses, pathogens tend to modify the cell envelope in order to prevent drugs from entering. This strategy, namely the reduction of membrane permeability, is a mechanism used by Gram-negative bacteria like *P. aeruginosa* and *A. baumannii*, where porin protein mutation limits antibiotic entry [35].

And perhaps the most ominous is the use of efflux pump systems: protein assemblies in bacterial membranes that actively export antibiotic molecules out. These pumps can confer cross-resistance to multiple antibiotic classes [36].

Lastly, the capacity of bacteria and yeasts to form biofilms on medical devices such as catheters or tubing on ventilators is a major obstacle to treatment. Within these self-generated, covering matrices, pathogens such as MRSA or *Candida* spp. can survive higher concentrations of antibiotics. In *A. baumannii*, for example, biofilms are made up of pili and polysaccharides regulated by specific genetic systems (e.g., BfmRS/Csu). This biofilm lifestyle enhances resistance further by shielding cells from antimicrobial action and facilitating gene transfer.

The net impact of these processes is to allow the emergence of multidrug-resistant (MDR) and even pan-drug-resistant (PDR) strains. This underlines the need for targeted antibiotic stewardship, novel antimicrobial agents such as efflux pump inhibitors, and stronger infection-control measures [36].

Although intrinsic molecular mechanisms of antimicrobial resistance, such as the production of inactivating enzymes, decreased membrane permeability, activation of efflux pumps for drugs, and biofilm formation, are constant regardless of the pandemic period, the COVID-19 crisis has also indirectly accelerated antibiotic resistance through factors related to human behavior. During the pandemic, numerous common practices have led to the widespread use of antibiotics, often even in the absence of confirmed bacterial co-infections [37]. For example, empirical prescription of broad-spectrum antibiotics was common in both intensive care and ordinary wards, as was prophylactic use to prevent secondary infections in high-risk patients and the concomitant administration of different classes of antibiotics. In addition, prolonged hospital stays, an overburdened healthcare system, staff shortages, and possible interruptions or malfunctions in infection control and antimicrobial stewardship programs have created an environment conducive to the selection and spread of multidrug-resistant pathogens. Overcrowding in hospitals, a lack of rapid diagnostic tests, and limited microbiological surveillance have further contributed to inappropriate prescribing. The factors described above may have favored the horizontal transfer of genes related to antibiotic resistance, particularly for organisms capable of creating biofilms, such as *Acinetobacter baumannii*, *Pseudomonas aeruginosa*, MRSA, and *Candida* spp. Overall, this suggests the urgent need to strengthen antibiotic stewardship by implementing targeted surveillance and applying rigorous infection control measures to mitigate the rapid emergence and spread of resistant pathogens during possible future global health crises [38].

## 5. Strategies for the Prevention of HAIs

**Figure 1:**
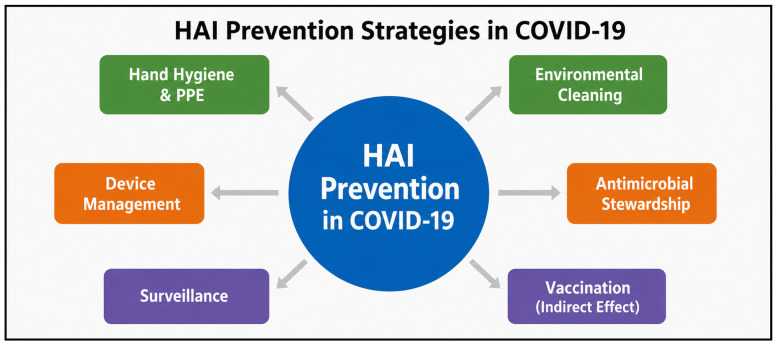
HAI prevention strategies in COVID-19.

The prevention of health care-associated infections (HAIs) during the COVID-19 pandemic requires a multidimensional approach, based on strong evidence and pre-existing protocols. During the COVID-19 pandemic, the prevention of HAIs required the implementation of measures adapted to the evolving healthcare context, with adaptations of standard procedures to address pandemic-related challenges. An observational study conducted in intensive care showed an almost double incidence of HAI in COVID-19 patients compared to non-COVID-19 patients, most of these were associated with mechanical ventilation [39]. Furthermore, data from the Center for Disease Control reported increases in CLABSI, CAUTI, VAE and bacteremia from MRSA in 2020 and 2021, closely corresponding to the peaks of hospitalization for COVID-19; at the same time, there was a reduction in cases of C. Difficile due to better hand hygiene and the use of personal protective equipment [40]. To this must be added the coexistence of structural factors such as staff shortages, intensive use of overtime, or hiring temporary staff which could contribute to the increase in HAIs [41]. These pandemic elements—the increased use of invasive devices, care pressure, disruptions in surveillance programs, and the overload of health systems—have highlighted the need to strengthen prevention measures (hand hygiene, environmental cleaning, device bundles, antimicrobial stewardship), in addition to standard practices. Against this background, the following strategies—both traditional and adapted to the pandemic scenario—have been implemented to reduce the burden of HAIs. Hand hygiene with proper use of personal protective equipment (PPE) remains the foundation of infection control. A systematic review points out that a strict implementation of hand hygiene guidelines—namely the WHO’s “Five Moments” approach—significantly lowers HAI rates [42,43]. The Geneva Hand Hygiene Model, which functioned from 1995 to 2000, achieved a significant reduction in overall hospital infections as well as MRSA transmission through a multimodal intervention that included staff education, monitoring, and ongoing feedback [44]. Sterilization and environmental sanitation are also crucial. Studies show that combining routine cleaning with high-tech disinfection modalities like hydrogen peroxide gas or ultraviolet devices significantly decreases contamination from resistant bacteria like MRSA and *Clostridioides difficile* [45,46]. Limiting the use and duration of invasive devices, such as central venous catheters, endotracheal tubes, and urinary catheters, is an essential preventive measure. Health care facilities using strict insertion and management guidelines have observed measurable decreases in catheter-associated bloodstream infections and CAUTIs. Active microbiologic surveillance also contributes to preventing infection, enabling early detection of outbreaks and resistant pathogens. Combined surveillance systems have been an important component in maximizing prevention policies and resource allocation. An important component of infection prevention and control is antimicrobial stewardship that seeks to optimize antibiotic use to avoid excessive exposure and slow the progression of resistance. Stewardship programs with strong emphasis on infections diagnosed promptly using rapid diagnostic tests, individualized treatment, and de-escalation strategies have repeatedly been associated with reduced antibiotic use without compromising patient safety [47,48]. Finally, a well-documented preventive strategy stems from care bundles, especially for ventilator-associated pneumonia (VAP). A meta-analysis of 22 studies had found that the use of a VAP bundle comprising head-of-bed elevation, oral care, subglottic suctioning, and cuff-pressure management had resulted in a reduction of more than 36% in the incidence of VAP, with reductions of above 65% and even to zero instances in some instances [49]. For example, in a prospective ICU study, the incidence of VAP dropped from 13.6 to 6.8 events per 1000 ventilator days after a modified bundle was introduced [50]. In brief, the combination of effective hand hygiene with appropriate strength, rigorous environmental cleaning, careful device handling, continuous microbiological surveillance, effective antimicrobial stewardship, and standardized care bundles has been associated with a significant reduction in HAIs. Especially in the heightened susceptibility of COVID-19 care, these measures are essential to reduce HAIs and improve patient outcomes. In terms of prevention strategies, mention must also be made of the vaccination campaign. Although vaccination acts to prevent SARS-CoV-2 infection and its progression to severe forms, its indirect contribution to mitigating HAIs must nevertheless be recognized. In fact, by reducing the number of severe cases of COVID-19 requiring hospitalization or even intensive care, vaccination campaigns have been able to reduce the burden on hospitals. This has reduced the need for invasive procedures such as mechanical ventilation and the placement of intravascular devices, which are well-known risk factors for HAIs. Furthermore, the reduction in intensive care admissions has limited the possibilities of colonization and transmission of multidrug-resistant organisms in high-risk environments. Moreover, by reducing the severity of COVID-19 cases, there has been a reduction in the length of hospital stays and the empirical use of broad-spectrum antibiotics, a known factor in antimicrobial resistance [51,52,53]. From a preventive standpoint, vaccination should be considered an integral part of a broad-based infection control strategy. Although it does not act directly on the nosocomial pathogen, reducing hospital overload and the use of devices indirectly supports the containment of HAIs. Ultimately, this underscores the importance of integrating immunization programs into public health safety initiatives. An integrated overview of the main prevention strategies for healthcare-associated infections in COVID-19 patients is illustrated in Figure 1.

## 6. Vaccination and Personal Protective Equipment in Healthcare Settings

The adoption of preventive measures by healthcare personnel is important to reduce the risk of SARS-CoV-2 infection and protect patients. The correct use of personal protective equipment (PPE) remains a key component. Recent studies have shown that non-compliance with PPE use is rare, but when present it can increase the risk of infection (IRR 1.3, IC 95% 0.8–2.3) [54]. The correct sequence of dressing and undressing is essential to avoid contamination. Specific guidelines, such as those from the CDC, and practical training of staff significantly reduce errors during undressing. In particular, face-to-face training has been found to be more effective than videos or written materials in preventing accidental contamination [55]. In terms of vaccination, coverage among Italian healthcare workers exceeded 80%, with a 77% reduction in the risk of infection (95% CI 70–82) [56]. Despite this, vaccine hesitancy persists in some groups, often related to perceptions of personal risk, trust in available information, and a sense of responsibility towards patients [57].

In summary, protecting staff and patients requires an integrated approach: correct use of PPE, effective vaccination, continuous training, and clear communication. Although the evidence is well established, practical challenges remain, such as stress management and long-term compliance, which must be addressed at the organizational level.

## 7. Discussion

The findings of the review highlight that management of healthcare-associated infections (HAIs) in COVID-19 patients needs to be an ongoing, multidisciplinary process. The emergence of co-infections and superinfections with multidrug-resistant (MDR) pathogens, as it frequently happens, also contributes significantly to therapeutic difficulty such that prompt microbiological diagnosis and targeted therapies are obligatory. Clinical trials during the pandemic once again established that diagnostic delays and longer empirical therapies are linked with increased mortality and additional spread of resistant strains [58,59,60,61,62].

Among the different HAIs, the increase in ventilator-associated pneumonia (VAP) and central line-associated bloodstream infections (CLABSIs) emerges as a consistent pattern in the available evidence, particularly in critically ill COVID-19 patients requiring intensive care and invasive support. In contrast, the pattern observed for catheter-associated urinary tract infections (CAUTIs) appears less consistent, with some data suggesting a reduction during the pandemic [7,8]. This variability may be related to differences in device utilization, changes in hospital admission patterns, and the reorganization of healthcare systems during the emergency.

Widespread administration of broad-spectrum antibiotics, frequently prescribed empirically to critically ill COVID-19 patients, has promoted the selection of resistant strains of *Klebsiella pneumoniae*, *Pseudomonas aeruginosa*, and *Acinetobacter baumannii*, with a cascade effect and catastrophic implications for infection control. Consistent with evidence presented by the World Health Organization (WHO) and the European Centre for Disease Prevention and Control (ECDC), antimicrobial resistance increased sharply during the pandemic, e.g., a drastic increase in carbapenemase-producing bacterial infections in intensive care units [63,64,65].

Healthcare-associated infections (HAIs) are highly prevalent in COVID-19 patients receiving ECMO and eCPR support, with incidence rates ranging from 36–68% of patients and 20–32 events per 1000 ECMO days. The most common infections are bloodstream infections (BSIs) and ventilator-associated pneumonia (VAP), though these infections do not consistently increase mortality in most studies [66,67,68,69].

The impacts of HAIs stretch from the clinical domain to public health policy, hospital resource utilization, and healthcare economics. HAIs account for prolonged hospital stay, expensive healthcare, and reduced availability of ICU beds at the height of pandemics. To face these challenges, hospitals should adopt novel surveillance systems based on quick diagnostics, artificial intelligence, and predictive epidemiological modeling [43]. Of the most important is the continuous education of health care staff. Regular updates in infection control policies, correct application of PPE, and standardized prevention practices—e.g., VAP bundles—have a key role in minimizing undesirable events. Clinical practice guidelines must be updated regularly to include evolving scientific evidence and epidemiological trends. Thus, to avoid HAIs in COVID-19 patients, both organizational and cultural transformations are needed that involve surveillance systems, antimicrobial stewardship programs, continuous staff education, and smarter utilization of hospital resources. Only an interprofessional response rooted in firm scientific evidence and interprofessional collaboration can potentially reduce the infection risk and enhance patient safety.

The COVID-19 pandemic has profoundly changed the epidemiological incidence, management methods and prevention techniques of HAIs; factors already mentioned such as the overload of health systems, staff shortages, the turnover of operators between the various departments, the prolonged use of invasive devices have allowed the creation of an unprecedented context, capable of promoting microbial spread. Current surveillance programs and antimicrobial stewardship have suffered a setback, with obvious delays in microbiological diagnoses and less adherence to infection control protocols. Several studies have been able to document the increase in infections associated with medical devices, such as CLABSI and VAP, as well as an increase in MDR pathogens such as carbapenem-resistant *Klebsiella pneumoniae* and *Acinetobacter baumannii*, often related to prolonged stays in intensive care and high exposure to antibiotics [39,41].

In addition, the mandatory use of personal protective equipment and isolation measures, although essential for the prevention of SARS-CoV-2 transmission, have sometimes reduced the frequency of patient monitoring, delaying the early recognition of signs of infection. The administration of broad-spectrum antibiotics on an empirical basis, in the unavailability of rapid diagnostics, has possibly further favored the increase in antimicrobial resistance [70,71,72].

During the COVID-19 pandemic, healthcare-associated infections (HAIs) in hospitalized patients have raised significant medico-legal and forensic concerns. A key challenge is distinguishing between unavoidable complications related to severe COVID-19 and preventable events associated with suboptimal care. In this context, forensic autopsies have proven essential, providing anatomical and histopathological evidence to clarify whether death resulted from disease progression or potential breaches in infection control practices [73,74].

International guidelines, including those from the College of American Pathologists (CAP) and the Royal College of Pathologists, emphasize the growing medico-legal importance of autopsies during the pandemic. They highlight the need for comprehensive documentation of comorbidities, infection status, and hospital-acquired pathogens, and encourage distinguishing deaths caused by SARS-CoV-2 from those related to its complications [73,75,76].

Furthermore, clinical autopsies have informed changes in clinical practice and supported the development of risk management strategies. They have helped identify patterns such as ventilator-associated pneumonia and central line-associated bloodstream infections linked to potential deviations in care, thereby promoting improvements in infection control practices and staff training [73,77].

From the forensic point of view, meticulous documentation and traceability of all diagnostic, therapeutic, and procedural actions are essential. Detailed medical records—handover documents, procedures with central lines or catheters, antibiotics administered, infection-control records—form objective evidence that will support or refute allegations of medical negligence. Courts use such documentation to determine whether or not the quality of care was attained [78,79,80].

Such documentation is not only essential for legal purposes but also represents a key component of clinical risk management, contributing to continuous quality improvement and safer care delivery. In addition, forensic practices during the pandemic have emphasized the importance of appropriate use of personal protective equipment (PPE), controlled environments, and systematic record-keeping from ICU admission to post-mortem evaluation, ensuring traceability and auditability of care processes [81,82].

Overall, the medico-legal relevance of HAIs in COVID-19 patients extends beyond clinical outcomes. Integrating autopsy findings with clinical data and infection control records can enhance transparency, improve patient safety, and reduce the risk of liability claims [83,84].

## 8. Limitations

This review presents some limitations that should be considered when interpreting the findings. As a narrative review, it does not provide the quantitative synthesis typical of systematic reviews or meta-analyses; however, a structured approach to the literature search and selection was adopted to enhance transparency and consistency. The available evidence is characterized by a certain degree of heterogeneity in study design, patient populations, healthcare settings, and diagnostic criteria, which may limit direct comparability across studies. In addition, most of the included studies are observational in nature, reflecting real-world clinical practice but potentially limiting the strength of causal inferences. Variations in local infection control practices, antimicrobial stewardship programs, and diagnostic capacities may also have influenced the reported findings. Finally, given the evolving nature of the COVID-19 pandemic and antimicrobial resistance patterns, some results may reflect specific phases of the pandemic and should be interpreted within this context.

## 9. Conclusions

The pandemic of COVID-19 has underscored the severe problems of healthcare-associated infections (HAIs), making a call for a comprehensive and forward-thinking strategy for infection prevention and control. An effective way forward must be built on several basic pillars.

First and foremost, rigorous infection prevention through standardized protocols is essential. Measures such as appropriate hand hygiene and the correct use of personal protective equipment, environmental cleaning and disinfection, careful management of invasive devices, active microbiological surveillance, and antimicrobial stewardship have all been shown to play a central role in reducing the burden of healthcare-associated infections. In addition, the implementation of standardized care bundles, particularly for ventilator-associated pneumonia, has been associated with significant improvements in infection control outcomes.

Second, rapid and accurate diagnostics are needed to enable early identification of pathogens and their resistance profiles. Molecular diagnostic platforms, PCR assays, and next-generation sequencing (NGS) have been critical to guide targeted antimicrobial therapy, reduce broad-spectrum antibiotic use, and improve patient outcomes. Timely diagnosis not only reduces time to effective treatment but also restricts the spread of multidrug-resistant organisms (MDROs) in healthcare facilities.

Third, prudent use of antimicrobials is necessary to halt the alarming rise of antimicrobial resistance (AMR). During the pandemic, empirical and often unnecessary antibiotic therapy—particularly for severe COVID-19 infection—led to increased selection pressure for resistant organisms, such as carbapenem-resistant *Klebsiella pneumoniae* and *Acinetobacter baumannii*. Antimicrobial stewardship programs, including de-escalation based on culture results, individualized dosing, and antibiotic course duration limitation, are critical in decreasing resistance without compromising patient safety.

Lastly, multidisciplinary collaboration among clinicians, microbiologists, infection preventionists, and clinical risk managers is paramount to addressing the complexity of HAIs. The multifaceted approach ensures that infection control is embedded within all stages of patient care, from ICU governance and surgery to hospital-wide surveillance and staff education.

In short, only through a combined and system-wide strategy—uniting prevention, advanced diagnostics, antimicrobial stewardship, and cross-disciplinary collaboration—can healthcare systems effectively reduce the effects of HAIs. By doing this, such a strategy will not only prevent morbidity and mortality but also enhance patient safety, quality of care, and hospital preparedness for future public health emergencies.

## Data Availability

No new data were created or analyzed in this study. Data sharing is not applicable to this article.
